# Supramolecular Gold Chemistry: From Atomically Precise Thiolate-Protected Gold Nanoclusters to Gold-Thiolate Nanostructures

**DOI:** 10.3390/nano10020377

**Published:** 2020-02-21

**Authors:** Rodolphe Antoine

**Affiliations:** Institut Lumière Matière UMR 5306, Université Claude Bernard Lyon 1, CNRS, Univ Lyon, F-69100 Villeurbanne, France; rodolphe.antoine@univ-lyon1.fr; Tel.: +33-(0)4-7243-1085

Supramolecular chemistry is defined as chemistry beyond the molecule. The supramolecular chemistry of gold and, in particular, of gold metalloligands leads to fascinating structural motifs with enhanced optical properties, as well as innovative catalytic activity. [1] The formation of a gold–sulfur bond is the driving force for the anchoring of thiol ligands on gold surfaces, as exemplified from self-assembled monolayers to nanoclusters (NCs) and nanoparticles (NPs) [2]. 

The chemistry of the gold–sulfur bond is extremely rich and leads to hybrid materials. Such materials encompass gold thiolate coordination oligomers, for instance [Au(I)(SR)]_n_, where SR stands for a chemical group containing a sulfur atom, and atomically well-defined clusters [Au_n_SR_m_], or supramolecular assemblies like Au(I)(SR) coordination polymers. While the majority of gold atoms in the nanoparticles are in the Au(0) state, under strong reducing conditions, gold atoms in supramolecular assemblies, like Au(I)(SR) coordination polymeric NPs, are in the gold(I) state. In atomically well-defined clusters of [Au_n_SR_m_] stoichiometry, the subtle balance between the Au(0) core and the Au(I)–SR shell leads to fascinating material properties and, in particular, to highly tunable optical properties.

The aim of this Special Issue on “Supramolecular Gold Chemistry” was to provide a unique international forum aimed at covering a broad description of results involving the supramolecular chemistry of gold with a special focus on the gold–sulfur interface leading to hybrid materials, ranging from gold–thiolate complexes, [3] to thiolate-protected gold nanoclusters [4,5,6,7,8,9,10,11] and gold–thiolate supramolecular assemblies or nanoparticles. [12,13,14] The role of thiolates on the structure and optical features of gold nanohybrid systems (ranging from plasmonic gold nanoparticles and fluorescent gold nanoclusters to self-assembled Au-containing thiolated coordination polymers) has been highlighted in the review article by Csapó and coworkers [14]. 

For gold–thiolate complexes and thiolate-protected gold nanoclusters, the atomically precise nature of their structures enables the elucidation of structure–property relationships, an essential step in their rational design for enhanced performances. From a theoretical point of view, the geometry of the clusters must be determined by quantum chemistry methods, and the optical responses described in terms of molecular transitions whose positions and intensities are predicted by sophisticated calculations of quantum mechanics. Bonačić-Koutecký and coworkers pioneered this concept and reported, in the early 1990s, the absorption spectra obtained with first-principle methods for the most stable structures of small bare metal clusters and nicely illustrated the molecular-like behavior of clusters, leading to an electronic energy quantization and changes in the leading features of the patterns as functions of the cluster sizes [15].

Structural characterization of nanoclusters is an active area of research and X-ray single-crystal diffraction has been the most straightforward and important technique in the structural determination of nanocluster nanomaterials in order to understand their structure–property relationships [16]. Not always applicable for nanoclusters, alternative approaches are to be explored. Separation techniques (liquid chromatography, gas phase ion mobility) can help in discriminating and characterizing structures. In this Special Issue, Antoine and coworkers combine an ion mobility-mass spectrometry approach with density functional theory (DFT) calculations for the determination of the structural and optical properties of gold thiolate oligomers (Au_10_(TGA)_10_ with TGA: thioglycolic acid) [3]. Whetten and coworkers combine electrospray ionization with high-performance liquid chromatography mass spectrometry (HPLC-MS) to separate and identify 3-MBA (MBA: mercaptobenzoic acid) protected gold nanoclusters, spanning a narrow size range from 13.4 to 18.1 kDa [5]. Theoretical investigations are also useful for structural characterization. Cheng and coworkers theoretically investigate Au_70_S_20_(PPh_3_)_12_ using density functional theory calculations. The electronic and geometric structure of Au_70_S_20_(PPh_3_)_12_ is further addressed based on the popular divide and protect concept and the superatom network model [7].

The discrete electronic states of nanoclusters cause molecular-like behavior, leading to fascinating physical–chemical properties, such as luminescence, magnetism, and catalysis, etc. Jin and coworkers highlight this molecular-like behavior by thoroughly exploring the differences in the photophysical properties of small organic molecules, gold–thiolate complexes, nanoclusters, and metallic-state nanoparticles [8]. The luminescence properties of 6-aza-2-thio-thymine stabilized gold nanoclusters [9] and gold thiolate coordination polymers [12] demonstrate the high potential of such nanomaterials for bio-sensing or lighting devices. However, in such nanosystems, the origin of photoluminescence (PL) is still not fully understood. Zhang and coworkers review some general PL mechanisms, from the pure metal-centered quantum confinement mechanism to the ligand-to-metal charge mechanism, as well as introducing a new paradigm, such as the ligand-centered p band intermediate state model [11]. On the other hand, gold nanoclusters have been proposed as a new, promising class of model catalyst [17]. Li and cowokers [6] and Negishi and coworkers [10] nicely review some interesting aspects of nanocluster catalysis for heterogeneous cross-coupling and for energy conversion.

Finally, synthetic routes are at the heart of supramolecular gold chemistry. Innovative strategies include a metal exchange reaction that leads to a new cluster compound in particular alloy nanoclusters. Zhu and coworkers describe a new type of metal exchange: self-alloying induced by intramolecular metal exchange, to produce the Ag_x_Au_25−x_(SR)_18_^−^ nanocluster [4]. Moreover, new synthetic routes, beyond wet chemistry using a reducing agent, are being explored. Pulsed laser ablation in liquids is such a new method, in which a solid target immersed in liquid is irradiated with a suitable pulsed laser beam. Kalarikkal and coworkers use this approach for the generation of 2D nanocomposites composed by gold nanoparticles and graphene oxide nanosheets [13]. Such new nanocomposites present remarkable chemical sensing for thiolates.

To conclude this overview on the papers published in the Special Issue “Supramolecular Gold Chemistry: From Atomically Precise Thiolate-Protected Gold Nanoclusters to Gold-Thiolate Nanostructures”, I am confident that the readers will enjoy these contributions and may be able to find inspiration for their own research within this Special Issue.
